# Measurement of ex vivo ELISpot interferon-gamma recall responses to *Plasmodium falciparum* AMA1 and CSP in Ghanaian adults with natural exposure to malaria

**DOI:** 10.1186/s12936-016-1098-8

**Published:** 2016-02-01

**Authors:** Harini Ganeshan, Kwadwo A. Kusi, Dorothy Anum, Michael R. Hollingdale, Bjoern Peters, Yohan Kim, John K. A. Tetteh, Michael F. Ofori, Ben A. Gyan, Kwadwo A. Koram, Jun Huang, Maria Belmonte, Jo Glenna Banania, Daniel Dodoo, Eileen Villasante, Martha Sedegah

**Affiliations:** Malaria Department, Naval Medical Research Center, Silver Spring, MD USA; Noguchi Memorial Institute for Medical Research, University of Ghana, Legon, Ghana; La Jolla Institute for Allergy and Immunology, La Jolla, San Diego, CA USA

**Keywords:** Circumsporozoite protein, CSP, Apical membrane antigen-1, AMA1, Natural transmission, Ghana, Ex vivo ELISpot, IFN-γ, T cells, Class I-restricted epitope, Peptide, HLA

## Abstract

**Background:**

Malaria eradication requires a concerted approach involving all available control tools, and an effective vaccine would complement these efforts. An effective malaria vaccine should be able to induce protective immune responses in a genetically diverse population. Identification of immunodominant T cell epitopes will assist in determining if candidate vaccines will be immunogenic in malaria-endemic areas. This study therefore investigated whether class I-restricted T cell epitopes of two leading malaria vaccine antigens, *Plasmodium falciparum* circumsporozoite protein (CSP) and apical membrane antigen-1 (AMA1), could recall T cell interferon-γ responses from naturally exposed subjects using ex vivo ELISpot assays.

**Methods:**

Thirty-five subjects aged between 24 and 43 years were recruited from a malaria-endemic urban community of Ghana in 2011, and their peripheral blood mononuclear cells (PBMCs) were tested in ELISpot IFN-γ assays against overlapping 15mer peptide pools spanning the entire CSP and AMA1 antigens, and 9–10mer peptide epitope mixtures that included previously identified and/or predicted human leukocyte antigen (HLA) class 1-restricted epitopes from same two antigens.

**Results:**

For CSP, 26 % of subjects responded to at least one of the nine 15mer peptide pools whilst 17 % responded to at least one of the five 9–10mer HLA-restricted epitope mixtures. For AMA1, 63 % of subjects responded to at least one of the 12 AMA1 15mer peptide pools and 51 % responded to at least one of the six 9–10mer HLA-restricted epitope mixtures. Following analysis of data from the two sets of peptide pools, along with bioinformatics predictions of class I-restricted epitopes and the HLA supertypes expressed by a subset of study subjects, peptide pools that may contain epitopes recognized by multiple HLA supertypes were identified. Collectively, these results suggest that natural transmission elicits ELISpot IFN-γ activities to class 1-restricted epitopes that are largely HLA-promiscuous.

**Conclusions:**

These results generally demonstrate that CSP and AMA1 peptides recalled ELISpot IFN-γ responses from naturally exposed individuals and that both CSP and AMA1 contain diverse class 1-restricted epitopes that are HLA-promiscuous and are widely recognized in this population.

**Electronic supplementary material:**

The online version of this article (doi:10.1186/s12936-016-1098-8) contains supplementary material, which is available to authorized users.

## Background

Despite the recent gains made in the battle against malaria, available control strategies may not be enough to ensure complete elimination and eradication [1]. Vaccines have been a cost-effective public health tool against many infectious diseases and the development of anti-malarial vaccines would be an important addition to existing malaria control strategies. The malaria parasite has a complex life cycle and it is believed that an effective anti-malarial sub-unit vaccine may need to target antigens in multiple stages of the parasite. Subjects in malaria-endemic areas develop partial immunity [2, 3] against severe disease and eventually against mild disease [4–6], and this supports the feasibility of developing vaccines against malaria. The role of antibodies to malaria antigens, especially the immunodominant circumsporozoite protein (CSP) but also other liver and blood stage antigens, has been widely investigated [6]. But whether naturally acquired antibodies to CSP [7, 8], or other antigens, provide protection against malaria remains elusive [6]. Sub-unit vaccines are being widely developed and the leading candidate, RTS, S which is based on the CSP, elicits protection in up to 50 % of subjects, but begins to wane after the first year [9, 10].

However, durable sterile immunity induced in humans by bites of radiation-attenuated *Plasmodium falciparum*-infected mosquitoes [11] or injection of purified radiation-attenuated sporozoites [12] is by far the strongest indicator of the feasibility of a malaria vaccine, and immunity is likely mediated by antibodies and T cell responses [12]. There is as yet no clearly defined correlate of protection against clinical malaria but the immune mechanisms mediating protection, at least in animal models, likely include interferon-γ (IFN-γ)-secreting CD8 + T cells that primarily target malaria antigens expressed in liver stages [13, 14]. Therefore, a second approach has been to use heterologous prime-boost regimens such as DNA/human adenovirus-5 that target the pre-erythrocytic stages using CSP and apical membrane antigen-1 (AMA1) [15, 16]. A DNA/Ad vaccine-induced sterile immunity in 27 % of subjects and CD8 + T cell IFN-γ responses to CSP and AMA1 contributed to protection [15, 16]. Protection was associated with class 1-restricted epitopes, particularly in AMA1 [15]. These and other class 1-restricted epitopes have been identified in CSP and AMA1 in subjects immunized with gene-based vaccines [17, 18] and radiation-attenuated sporozoites [19, 20] as well as in subjects in malaria-endemic areas [21–23].

As with antibodies, the role of naturally acquired T cell responses in providing protection against malaria infection or disease remains largely unanswered., although ELISpot IFN-gamma (IFN-γ) has been used to measure naturally acquired CD8 + T cell responses [6, 24]. Recently, in a study with subjects who were naturally exposed to *P. falciparum* malaria in Ghana [25], class 1-restricted T cell IFN-γ responses were successfully measured using ex vivo ELISpot. A major finding of this earlier study was that peptides containing predicted class 1-restricted epitopes recalled ELISpot responses from human leukocyte antigen (HLA)-matched subjects, although the frequencies of such responses were lower than recall responses induced by longer HLA DR-restricted peptides [25]. However, since the magnitude of responses was generally low, it was essential to use an appropriate definition of positivity that reproducibly distinguished antigen-specific ELISpot activities induced by natural exposure without vaccine intervention. On this basis, a definition of lower stringency (at least a doubling of spot-forming cells/million (sfc/m) peripheral blood mononuclear cells (PBMC) in test wells relative to control wells, and a difference of at least 10 sfc/m between test and control wells) than that used in vaccine trials conducted by the Naval Medical Research Center (NMRC) [26] was effective for assessing positivity of ex vivo ELISpot IFN-γ responses to *P. falciparum* CSP and AMA1 in naturally exposed subjects [25]. Of the number of positive responses identified using this stringency definition, 82 % remained positive when the positivity definition used for vaccine-induced responses at NMRC [26] was applied. This positivity definition was adapted for a study measuring naturally acquired ELISpot IFN-γ responses to the *P. falciparum* cell-traversal protein for ookinetes and sporozoites (CelTOS) in Ghana, namely a stimulation index of >2.0 (response obtained with PBMCs stimulated with malaria antigen peptides compared to medium alone) and a difference of 10 sfc/m between antigen-stimulated and unstimulated PBMCs [27].

These positivity criteria were applied in the current study to better determine the potency of 15mer peptides spanning the entire sequences of the vaccine candidate antigens CSP (3D7) and AMA1 (3D7) for induction of IFN-γ recall responses in PBMCs from naturally exposed subjects in Ghana using ex vivo ELISpot assays. Peptide pools containing 9–10mers that represent known or predicted class 1-restricted epitopes and grouped according to HLA supertypes were also tested against subject PBMCs. This was to assess the feasibility of using 9–10mer pools to predict the HLA restriction of responses to the 15mer overlapping peptides since this would provide further insights into the genetic restriction of naturally acquired ex vivo ELISpot IFN-γ responses to CSP and AMA1 in the same population. Since 11 of the 35 tested subjects had been HLA-typed, it was possible to determine whether such HLA-restricted pools would detect matched HLA-restricted responses of HLA-typed subjects. For subjects who were not HLA-typed, it was possible to use these HLA pools to examine the HLA-restriction of positive responses in some subjects, and the observed activities could be best explained by the promiscuity of class I-restricted epitopes [19, 28–30]. Taken together, the results obtained from these analyses provide evidence that a malaria vaccine containing CSP and AMA1 may induce broad immune responses in a genetically diverse population. These results also demonstrate the need to include field assessment of HLA-restricted epitopes in malaria vaccine design strategies.

## Methods

### Ethics

This study was conducted according to the human research protocol ‘Quality Control of Immunological Reagents and Validation of Improvements to Immunological Assays in Support of Malaria Vaccine Trials’, which was approved by Institutional Review Boards at the Noguchi Memorial Institute for Medical Research (NMIMR) and the NMRC. NMIMR holds a US Government Federal-wide Assurance (FWAA00001824) from the Office for Human Research Protections, as does NMRC (FWA00000152). NMRC also holds a Department of Navy Addendum to the FWA for human subject protections (DoDI 3216.02). The protocol was conducted in accordance with the principles in The Belmont Report and federal regulations regarding the protection of human subjects in research including 32 CFR 219 (The Common Rule), and all regulations pertinent to the Department of Defense, the Department of the Navy, the Bureau of Medicine and Surgery of the US Navy and internal NMRC policies for human subject protections and responsible conduct of research. All NMRC and NMIMR personnel contributing to or performing human research were certified as having completed human research ethics education and training. Written informed consent was sought from all study subjects who willingly agreed to be part of the study and met the inclusion criteria.

### Study site

The study was conducted within the University of Ghana, Legon and its surrounding communities in Accra, Ghana. Legon is about 10 km north of Accra, the capital city of Ghana. It is home to the University of Ghana, and a 10 sq km area around Legon has an approximate population of 100,000. Malaria transmission is very low and limited mainly to the rainy season from March to November.

### Participants

Study subjects were male and female adults between 24 and 43 years (average age 29 years) who were resident in the study area. Eligibility criteria for the study were the following: age 18–55 years; males, or females who were not pregnant or nursing; normal screening medical history and physical examination; haemoglobin >10 g/dL; absence of known immunodeficiency (>400 CD4 + T cells/μL); and, negative hepatitis B and C serology. All participants generally had a normal medical history at screening and physical examination. A total of 45 subjects were screened and 35 who met the inclusion criteria were included in the study. The 35 selected study subjects were subsequently screened for malaria parasites by rapid diagnostic test (RDT) kits and by light microscopy.

### Sample collection

Sixty ml of venous blood was collected per subject into heparinized tubes. PBMCs were isolated from blood by gradient centrifugation using Accuspin Histopaque-1077 cell separating tubes. After washing and counting, cells were rested in an incubator at 37 °C, 5 % CO_2_ for a maximum of 20 h before use in ex vivo ELISpot assays.

### HLA typing

For 11 of the 35 subjects, low-moderate resolution HLA typing for HLA-A and HLA-B was conducted by the Department of Defence Bone Marrow Donor Programme using the ABDR SSP Unitray system (Pel-Freeze, Brown Deer, WI, USA) according to the manufacturer’s instructions, and as used previously [25].

### Synthetic peptides and peptide pools

Ex vivo ELISpot IFN-γ assays used commercially synthesized 15mer peptides that overlapped by 11 amino acids (Chiron Technologies, Clayton, Victoria, Australia). These 15mer peptides represented the full-length CSP (397 amino acids) and AMA1 (622 amino acids) antigens, each from strain 3D7 [25]. These were combined into nine pools for CSP (Cp1-Cp9) containing three to 12 peptides per pool, and 12 pools for AMA1 (Ap1-Ap12) containing ten to 13 15mer peptides per pool (Additional file 1: Table S1 and Additional file 2: Table S2). Class 1-restricted HLA-binding 9–10mer peptides within CSP and AMA1 were predicted using NetMHC [31] or as previously published [17, 18] and were defined according to their supertype classification [32]. The choice of these HLA-binding peptides was on the basis that their predicted HLA supertypes are among the most globally prevalent HLA alleles [32]. The HLA-binding peptides were synthesized (Alpha Diagnostics Intl Inc, San Antonio, TX, USA, (>91 % purity)) and grouped into peptide pools containing predicted 9–10mer peptides for each HLA supertype. Details of the mixtures of predicted 9–10mer HLA-binding pools from the CSP and AMA1 antigens, and the CSP or AMA1 15mer peptide pools in which the corresponding 9–10mer epitopes were contained, are presented in Table 1 (CSP) and Table 2 (AMA1), respectively. The locations of these epitopes within the 15mer peptides are shown (in italics and bolditalics) in Additional file 1: Table S1 and Additional file 2: Table S2. All peptides were originally in sterile plain RPMI and were diluted to the required concentration with RPMI 1640 with 1 % penicillin–streptomycin, 1 % l-glutamine and 10 % normal human serum) before use.

**Table 1:** Peptide composition of the five predicted CSP HLA A- and B-binding peptide pools

15mer pool	C-HLA A01	C-HLA A02	C-HLA A03	C-HLA A24	C-HLA B27
Cp1	*FVEALFQEY* ^a^ *(B07)* ^a^ *LFVEALFQEY* ^a^ (B07)e (A01, B44)^f^ (A03,)^f^ EALFQEYQC^e^ (B27)^f^	*SVSSFLFVEA* ^a^ (*A02* ^a^, A30)^f^ *FLFVEALFQE* ^a, f^ *(A01A24)* ^a^ *ILSVSSFLFV* ^b^ (A24)^e^ (A02)^f^ *MMRKLAILSV* ^c, e^(A01A03)^e^	QCYGSSSNTR^e^ (B27)^f^ CYGSSSNTR^e^ (B27)^f^	*SFLFVEALF* ^a^ (A01A24, A24)^e, f^ *AILSVSSFLF* ^a^ (B07)^e^ (B58)^f^	LAILSVSSF^e^ (B07, B27, B62)^e^ (B27)^f^ ALFQEYQCY^e^ (A01, B27, B62)^e^ YQCYGSSSN^e^ (B62)^e^ (B27)^f^ SSFLFVEAL^e^ (B62)^e^ (A01)^e^
Cp2					
Cp3					
Cp4					
Cp5					
Cp6	*EPSDKHIKEY* ^b^ (B07, B44)^f^	*YLNKIQNSL* ^a, d^ (A01, B08)^e^		*EYLNKIQNSL* ^b^ (A02, A24, B08, B27)^e^ (A02, B27)^f^	IQNSLSTEW^e^ (B27, B62)^e^ (B07, B27)^f^
Cp7		SVTCGNGIQV^e, f^	*VTCGNGIQVR* ^b^		IQNSLSTEW^e^ (B27, B62)^e^ (B07, B27)^f^
Cp8			ELDYANDIEK^g^ (A01)^e^		
Cp9		*SVFNVVNSSI* ^a, f^ *(A24)* ^a^ (B27)^f^ NVVNSSIGLI^a, f^ *GLIMVLSFL* ^b, f^	ELDYANDIEK^g^ (A01)^e^		KMEKCSSVF^e^ (A01, B27, B62)^e^ (B44)^f^ *IMVLSFLFL* ^e^ *(A02)* ^a^ (A24, B27)^f^ *LIMVLSFLF* ^a^ (*A01A24*)^a^ (A01, A24, B27, B62)^e^ (A24, B27)^f^

Peptides (9–10mers) representing HLA supertypes A01, A02, A03, A24, and B27 were grouped into five pools. The corresponding Cp pool containing each epitope is also shown. Allele groups are assigned to supertypes where known. HLA-restrictions were defined either by testing with HLA-matched subjects or predicted using NetMHC. Epitopes that experimentally recalled responses from subjects either immunized with malaria vaccines or exposed to malaria transmission are shown in italics

^a^Reference [17]

^b^References [19, 20]

^c^Reference [21]

^d^References [22, 23]

^e^predicted by NetMHC for US subjects

^f^predicted by NetMHC for Ghanaian subjects

^g^Epitope is in 15mer peptides that are contained in Cp8 and Cp9

**Table 2:** Peptide composition of the six predicted AMA1 HLA A- and B-binding peptide pools

15mer pool	HLA A01	HLA A02	HLA A03	HLA A24	HLA B07	HLA B44
Ap1	LLSAFEFTY^b^ (B07)^b^ (A03, B58)^c^ GQNYWEHPY^b^ (B27)^c^	KLYCVLLLSA^b^ (A03)^b^ (A02, A03, A30)^c^ SAFEFTYMI^b^ (A01)^b^	EFTYMINFGR^b^ (A03)^c^ DVYRPINEHR^b, c^ (A03)^c^	LYCVLLLSAF^b^ (B07)^b^	YPLHQEHTY^b, c^	*FEFTYMINF* ^a, c^ (B27)^c^ *YEYPLHQEH* ^*a*, c^ (B27)^c^
Ap2	NYMGNPWTEY^b^ (B07, B44)^b^ (A01A24, B27)^c^	NLFSSIEIV^b, c^ YMGNPWTEYM^b, c^ (A01, B44)^b^ (B27)^c^	LFSSIEIVER^b, c^			DENTLQHAY^b, c^ (A01, B07)^b^ QEQNLFSSI^b^ (B27)^c^ IEIVERSNY^b, c^
Ap3		YMAKYDIEEV^b, c^ (B27)^c^	TQYRLPSGK^b, c^ (A34, A01A03)^c^			
Ap4	*TLDEMRHFY* ^a^ (B07)^b^ (A03, A01)^c^ *MRHFYKDNKY* ^a^ (B27)^c^ LTPVATGNQY^b^		MSPMTLDEMR^b^ (B07)^b^ TLDEMRHFYK^b^ (A01)^b^ (A01)^c^	QYLKDGGFAF^b^ (A01, A02, B07, B44)^c^		TEPLMSPMTL^b^ (B07)^b^
Ap6	DISFQNYTY^b^ (B07)^b^ (B27, B58)^c^		NSMFCFRPAK^b, c^ (A02)^b, c^ (A03, A01A03, A30, B27)^c^ SFQNYTYLSK^b^		CPRKNLQNA^b, c^	
Ap7	LSASDQPKQY^b^ (A01, B27, B58)^c^				IPHVNEFPA^b, c^	*NEFPAIDLF* ^a, c^ FECNKLVFEL^b, c^ (A02, B44, B27)^c^
Ap8	HGKGYNWGNY^b^ (A30, B27)^c^	*TQKCEIFNV* ^a^ (B27)^c^ FLPTGAFKA^b, c^ ETQKCEIFNV^a, c^ CLINNSSYI^a^ (B27)^c^	RYKSHGKGY^b^ (A01)^b^ (A01A03, A30, B27)^c^ SAFLPTGAFK^b, c^ (B07)^b^ (B27)^c^			TETQKCEIF^b^
Ap9	VENNFPCSLY^b^ (B44)^b^ (B27, B44)^c^	CLINNSSYI^b, c^ (B27)^c^	SLYKDEIMK^b, c^ (B27)^c^ (B27, A01A03)^c^		FPCSLYKDEI^b^ HPIEVENNF^b, c^	VENNFPCSL^b^ VENNFPCSLY^b, c^ (A01)^b^ (B27, B44)^c^
Ap10		EVTSNNEVVV^b, c^ IIIASSAAV^b, c^	FISDDKDSLK^b, c^ (A02)^b,c^ RFFVCKCVER^b, c^		KPTYDKMKII^b, c^	*NEVVVKEEY* ^a, c^
Ap11		AVLATILMV^b, c^ VLATILMVYL^b, c^ (A01, A03)^b^ (A30)^c^	VLATILMVY^b^ (A01)^c^ TILMVYLYKR^b^ ILMVYLYKRK^b^ (A02)^b^ (A03)^c^			
Ap12	TTPVLMEKPY^b^ (B07)		EASFWGEEKR^b^ (A34)^c^ HTTPVLMEK^b, c^		RASHTTPVL^b^ (A02, B07, B27, B58)^c^	

Peptides (9-10mers) representing HLA supertypes A01, A02, A03, A24, B07 and B44 were grouped into six pools. The corresponding Ap pool containing each epitope is also shown. HLA-restrictions were defined either by testing with HLA-matched subjects (Reference 16) or predicted using NetMHC. Allele groups are assigned to supertypes where known, or are listed as individual alleles. Epitopes that experimentally recalled responses from subjects either immunized with malaria vaccines or exposed to malaria transmission are shown in *bold*

^a^Reference [18]

^b^Predicted by NetMHC for US subjects

^c^Predicted by NetMHC for Ghanaian subjects

### Ex vivo ELISpot IFN-γ assays

ELISpot IFN-γ assays were performed as previously described [33]. Briefly, multiscreen plates (Millipore Corporation, USA) were coated with 100 µl/well of 15 µg/ml anti-human IFN-γ monoclonal antibodies (Mabtech AB, USA) in 0.1 M bicarbonate buffer, pH 9.6. Plates were incubated at 4 °C overnight, washed six times with RPMI 1640 and blocked for at least 2 h with assay blocking buffer. Plates were subsequently washed as described above and PBMCs (400,000 cells/well) from each subject were tested in duplicate with all CSP and AMA1 15mer peptide pools as well as with the pools of predicted HLA A- and B-specific peptide pools (10 μg/ml of each peptide in all pools). Concanavalin A (Con A, Sigma Aldrich, USA) (0.625 μg/ml) and CEF (Cellular Technology Ltd, USA) (2.0 μg/ml) were positive controls in all assays. Subject PBMCs incubated with medium only were used as negative controls. After PBMC incubation for 36 h at 37 °C, 5 % CO_2_, plates were washed six times with 250 μl/well of wash buffer (PBS containing 0.05 % Tween 20) and incubated with 100 μl/well of 1 μg/ml biotinylated anti-IFN-γ monoclonal antibody (Mabtech, USA) diluted in 0.5 % fetal calf serum (FCS) in PBS for 3 h at room temperature. Plates were again washed six times and incubated with 100 μl/well of 1 μg/ml alkaline-phosphatase-conjugated streptavidin (Mabtech, USA) for 1 h at room temperature. Plates were afterwards washed six times as above and three times with plain PBS before incubation with an enzyme-specific chromogenic substrate (Bio-Rad, USA) for 15 min at room temperature. Colour development was terminated by washing of plates under tap water and the plates air-dried at room temperature. The number of IFN-γ-producing cells in the form of spots per well was subsequently estimated using an automated ELISpot plate reader (AID GmbH, Germany) and the acquired data was exported into Microsoft Excel for analysis.

### Data analysis

Activities were calculated as sfc/m PBMCs. The assay was considered positive if there was (1) at least a doubling of sfc/m in test wells relative to control wells, and (2) a difference of at least ten spots between test and control wells. This definition was adapted for use in a recent study that evaluated a different malaria antigen, CelTOS [27]. Subjects were considered positive to a malaria antigen if their PBMCs tested positive against at least one peptide pool. All graphics were created in Microsoft Excel.

## Results

Forty-five healthy Ghanaian adults were screened for this study, and 35 subjects between 24 and 43 years (average age of 29 years) who met eligibility requirements and gave informed consent participated in the study. All subjects were negative for malaria by light microscopy and malaria RDTs and all female subjects were not pregnant. For each subject, the ELISpot activity (sfc/m) for the unstimulated medium control was subtracted from the activities (sfc/m) for each test peptide. All subjects in this study made positive IFN-γ responses to Con A or CEF or both, as previously reported by Anum et al. [27]. In all assays, unstimulated medium control responses ranged between 0 and 18 sfc/m except subjects v15 and v28 whose mean unstimulated medium responses were 69 and 88 sfc/m respectively.

### Ex vivo ELISpot IFN-γ responses to CSP overlapping peptide pools

**Fig. 1:**
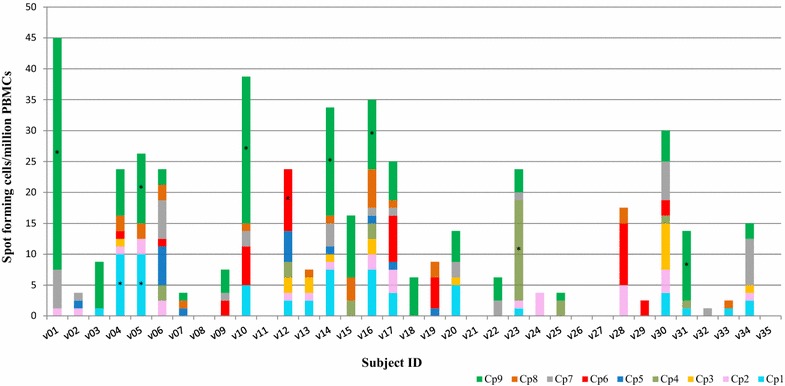
Number of IFN-γ-secreting cells in response to stimulation with the nine 15mer CSP peptide pools. Values are expressed as spot forming cells per million PBMCs. *Stacked bars* represent responses to the different Cp pools and *asterisks* indicate responses that met the positivity criteria as described in (“Methods”) section

Nine of 35 subjects (26 %) were positive with at least one CSP 15mer peptide pool (Fig. 1, Table 3). Of these, eight subjects were positive to a single 15mer pool and one subject was positive to two CSP 15mer pools. The highest number of positive responses was to Cp9 (six subjects), followed by Cp1 (two subjects), and Cp4 and Cp6 (one subject each) (Fig. 1). The highest CSP 15mer pool activities were to Cp9 (v01: 38 sfc/m, v10: 24 sfc/m, v14: 18 sfc/m) and Cp4 (v23: 16 sfc/m). None of the subjects made positive responses to the CSP 15mer peptide pools Cp2, Cp3, Cp5, Cp7, and Cp8. Also, 26 subjects did not respond to any of the nine CSP peptide pools (Fig. 1), even though all subjects responded to the positive controls; either Con A or CEF or both [27]. Thirteen CSP negative subjects were positive with AMA1 15mer pools.

**Table 3:** Activities to CSP peptide pools and CSP HLA peptide pools—matching activities of peptide pools

Subject	HLA supertype	CSP 15mer pool	CSP HLA pool	Interpretation
HLA-typed				
v01	**A01 A03 B27 B27**	Cp9	C-HLA **A03**	***A03*** Matched^a^ A03 pool contains promiscuous ***A01*** and ***B27*** epitopes^b^
v02	**A02 A03 B27 B44**	NEG	C-HLA A01, C-HLA **A02,** C-HLA **A03**	***A02***, ***A03*** Matched^a^ A01 pool contains promiscuous ***A03***, ***B27*** and ***B44*** epitopes^b^ A02 pool contains promiscuous ***B27*** epitope^b^ A03 pool contains promiscuous ***B27*** epitopes^b^
v04	**A01 A24 B27 B27**	Cp1	NEG	Cp1 pool contains matched identified ***A01***, ***A24*** and ***B27*** epitopes^c^ Cp1 pool contains matched promiscuous ***A01***, ***A24*** and ***B27*** epitopes^d^
v05	**A03** A01A03 **B27 B44**	Cp1, Cp9	NEG	Cp1 pool contains matched identified ***A03*** and ***B27*** epitopes^c^ and promiscuous ***A03*** and ***B44*** epitopes^d^ Cp9 pool contains matched identified ***B27*** and promiscuous ***B44*** epitopes^d^
v10	**A02 A02** B07 B07	Cp9	NEG	Cp9 pool contains matched ***A02*** epitopes^c^
v12	A01A24 A03 **B07 B27**	Cp6	NEG	Cp6 pool contains promiscuous ***B07*** and ***B27*** epitopes^d^
v16	**A03 A03 B27 B58**	Cp9	C-HLA A01	A01 pool contains promiscuous ***A03*** and ***B27*** epitopes^b^ Cp9 pool contains promiscuous ***A01***, ***B27*** and ***B58*** epitopes^d^
HLA not typed				
v13		NEG	C-HLA A24	Probably ***A24*** matched^b^ A24 pool contains promiscuous A02, B07, B27 and B58 epitopes^b^
v14		Cp9	C-HLA **A01**, C-HLA **A02**, C-HLA **A24**	Probably ***A02*** matched as Cp9 contains identified A02 epitopes^a,c^ Cp9 pool contains promiscuous ***A01*** and ***A24*** epitopes^d^
v30		NEG	C-HLA B27	Probably ***B27*** matched^a^ B27 pool contains identified promiscuous A01A24 and A02 epitopes, and predicted A01, A24, , B07, B58 and B62 epitopes^b^
v23		Cp4	NEG	No HLA prediction possible^e^
v31		Cp9	NEG	No HLA prediction possible^e^
No. positive		9 (26%)	6 (17%)	
Total Positive	12 (34%)			

Activities of subjects to either CSP 15 mer peptide pools or CSP HLA peptide pools are shown; subjects that were negative with both sets of peptide pools were excluded. For each subject, the HLA supertypes that are matched by the tested peptides are italicized in bold and underlined, while predicted promiscuous epitopes are italicized and in bold. Positivity was defined as described in “Methods”

^a^Matched, where activity of a CSP HLA pool matched that subject’s HLA

^b^Matched through analysis of promiscuous epitopes in that positive CSP HLA pool and that match subject’s HLA

^c^Matched through analysis of predicted epitopes in that positive CSP pool that match that subject’s HLA

^d^Matched through analysis of promiscuous epitopes in that positive CSP pool that match that subject’s HLA

^e^In the absence of positive HLA peptide pools, no HLA prediction is possible

### Ex vivo ELISpot IFN-γ responses to predicted HLA-binding CSP peptide pools

**Fig. 2:**
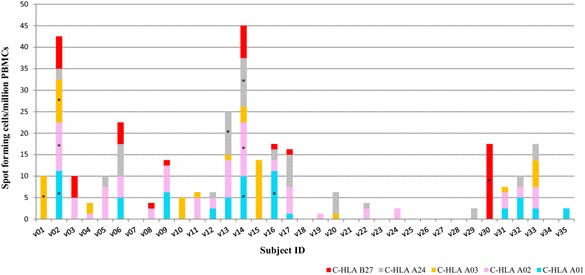
Number of IFN-γ-secreting cells in response to stimulation with the five CSP HLA pools. Values are expressed as spot forming cells per million PBMCs. *Stacked bars* represent responses to the different C-HLA pools and *asterisks* indicate responses that met the positivity criteria

Six of the 35 subjects (17 %) responded positively to at least one of the CSP predicted HLA-binding peptide pools tested (Fig. 2, Table 3). Three of these subjects (v01, v02 and v16) were HLA-typed while the other three (v13, v14 and v30) were not. Of the three HLA-typed subjects, two were positive to a single HLA pool (v01 to the A03 pool and v16 to the A01 pool) while the third (v02) was positive to three HLA pools (restricted by A01, A02 and A03) (Table 3). For the three subjects with unknown HLA types, two were positive to a single HLA pool (v13 to the A24 pool and v30 to the B27 pool) while the third (v14) was positive to three HLA pools (restricted by A01, A02 and A24) (Table 3).

For all six positive subjects the highest number of positive responses was to HLA A01 pool (three subjects), HLA A02, HLA A03 and HLA A24 pools (two subjects), and HLA B27 pool (one subject) (Fig. 2). Thus, there were positive responses to all five HLA-binding CSP peptide pools among the six responders.

### Interpretation of responses to CSP 15mer peptide pools and HLA pools

Seven HLA-typed and five non-HLA-typed subjects were positive with either CSP 15mer pools or C-HLA pools or both (Table 3). Three of the HLA-typed subjects were positive with C-HLA pools (Table 3); two of these subjects were positive with C-HLA pools that contained HLA-matched epitopes and one subject was positive with a C-HLA pool (A01) that contains promiscuous epitopes restricted by the subject’s matching HLA (A03). Four HLA-typed subjects were positive with CSP 15mer pools but negative with C-HLA pools, and these CSP 15mer pools contain HLA matched epitopes or promiscuous HLA matching epitopes. This interpretation can be applied to three of the five non-HLA-typed subjects who were positive with matched and promiscuous epitopes. No prediction was possible for the remaining two positive but non-HLA-typed subjects.

### Ex vivo ELISpot IFN-γ responses to the AMA1 peptide pools

**Fig. 3:**
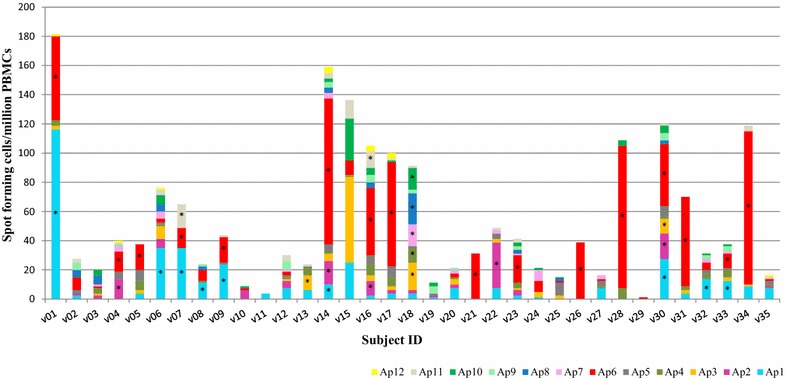
Number of IFN-γ-secreting cells in response to stimulation with the 12 15mer AMA1 peptide pools. Values are expressed as spot forming cells per million PBMCs. *Stacked bars* represent responses to the different Ap pools and *asterisks* indicate responses that met the positivity criteria

Twenty-two of 35 subjects (63 %) responded positively to at least one of the AMA1 15mer peptide pool (Fig. 3, Table 4), and therefore the frequency of responses to AMA1 was greater than to CSP (26 %). Of these, 13 subjects were positive to one AMA1 15mer peptide pool, four subjects were positive to two peptide pools, three subjects were positive to three peptide pools, one subject was positive to four peptide pools, and one subject was positive to five peptide pools (Fig. 3). The highest number of positive responses was to Ap6 (16 subjects), Ap1 (nine subjects) and Ap2 (five subjects). The highest AMA1 15mer pool activities were to Ap1 (v01: 116 sfc/m) and Ap6 (v01: 58 sfc/m, v14: 100 sfc/m, v17: 71 sfc/m, v28: 98 sfc/m, v31: 61 sfc/m and v34: 105 sfc/m). This further shows that, aside response frequencies, the magnitude of responses to AMA1 15mer peptides was also higher than that to CSP 15mer peptides since none of the CSP peptide responses was greater than 50 sfc/m. None of the AMA1 positive subjects had positive responses to pools Ap5, Ap9 and Ap12, and 13 subjects did not respond to any of the 12 AMA1 peptide pools (Fig. 3). Two AMA1 negative subjects (v10 and v12) were positive with CSP pools. The unstimulated PBMC control from one subject (v15) had high ELISpot IFN-γ responses and this subject’s responses to almost all stimulants did not meet the positivity criteria despite having apparently high activities (Figs. 1, 3).

**Table 4:** Activities to AMA1 peptide pools and AMA1 HLA peptide pools—matching activities of peptide pools

Subject	HLA supertype	AMA1 15mer pool	AMA1 HLA pool	Interpretation
HLA-typed				
v01	**A01 A03 B27 B27**	Ap1, Ap6	**A01**, B44	***A01*** Matched^a^ B44 pool contains promiscuous ***A01*** and ***B27*** epitopes^b^ A01 pool contains promiscuous ***A03*** and ***B27*** epitopes^b^ Ap1 and Ap6 pools contain promiscuous ***A01***, ***A03*** and ***B27*** epitopes^d^
v02	**A02 A03** B27 **B44**	NEG	**A02**	***A02*** Matched^a^ A02 contains promiscuous ***A01, A03*** and ***B44*** epitopes^d^
v03	**A01 A03** B07 B58	NEG	A02	A02 pool contains promiscuous ***A01*** and ***A03*** epitopes^b^
v04	**A01** A24 **B27 B27**	Ap2, Ap6	NEG	Ap2 pool contains matching ***A01*** epitopes^c^ Ap6 pool contains matching ***A01*** epitopes^c^, and promiscuous ***B27*** epitopes^d^
v05	**A03** **A01A03 B27** B44	Ap6	**A03**	***A03*** Matched^a^ A03 contains promiscuous ***A01A03***, ***A03*** and ***B27*** epitopes^d^
v08	A01A03 **A02 B27 B44**	Ap1	NEG	Ap1 pool contains matching ***A02*** and ***B44*** epitopes^c^, and promiscuous ***B27*** epitopes^d^
v10	**A02 A02 B07 B07**	NEG	**A02**, A03	***A02*** matched^a^ A03 pool contains promiscuous ***A02*** and ***B07*** epitopes^d^
v12	A01A24 **A03** B07 **B27**	NEG	A02	A02 pool contains promiscuous ***A03*** and ***B27*** epitopes^d^
v16	**A03 A03 B27 B58**	Ap2, Ap6, Ap11	A02	A02 contains promiscuous ***A03*** and ***B27*** epitopes^d^ Ap6 contains promiscuous ***A03, B27*** and ***B58*** epitopes^d^
v17	**A01A03 A03 B07 B44**	Ap6	NEG	Ap6 contains matching ***A03*** and ***B07*** epitopes^c^, and promiscuous ***A01A03*** and ***B07*** epitopes^d^
v19	**A03** A24 B44 B62	NEG	**A03**	***A03*** Matched^a^
HLA not typed				
v06		Ap1	**A02, A03**	Ap1 pool contains matching ***A02*** and ***A03*** epitopes^e^
v09		Ap1, Ap6	**B07**	Ap1 and Ap6 pools contain matching ***B07*** epitopes^e^
v13		Ap7	A24	Ap7 and A24 contains promiscuous ***A01***, ***A02*** *and* ***B44*** epitopes^e,f^
v14		Ap1, Ap2, Ap6	**A01, A02, A03, A24, B07**	Ap1 pool contains matching ***A01***, ***A02***, ***A03***, ***A24***, ***B07*** and ***B44*** epitopes^e^ Ap2 contains matching ***A01***, ***A02*** and ***A03*** epitopes^e^, and promiscuous ***B07*** epitopes^f^ Ap6 contains matching ***A01***, ***A03*** and ***B07*** epitopes^5^, and promiscuous ***B07*** epitopes^f^
v22		Ap6	**A03**	Ap6 pool contains matching ***A03*** epitopes^e^
v30		Ap1, Ap2, Ap3, Ap6	**A03, B07**	Ap1, Ap2, Ap3 and Ap6 pools contain matching ***A03*** epitopes^e^ Ap1 and Ap6 pools contain matching ***B07*** epitopes^e^
v31		Ap6	**A03**	Ap6 pool contains matching ***A03*** epitopes^e^
v32		Ap1	**A03**	Ap1 pool contains matching ***A03*** epitopes^e^
v33		Ap1, Ap6	**A03**	Ap1 and Ap6 pools contain matching ***A03*** epitopes^e^
v18		Ap3, Ap4, Ap7, Ap8, Ap10	NEG	No prediction possible^g^
v07		Ap1, Ap6, Ap11	NEG	No prediction possible^g^
v20		NEG	A03	Probably contains ***A03***-matched epitopes^e^
v21		Ap2	NEG	No prediction possible^g^
v23		Ap6	NEG	No prediction possible^g^
v26		Ap6	NEG	No prediction possible^g^
v28		Ap6	NEG	No prediction possible^g^
v34		Ap6	NEG	No prediction possible^g^
No. positive		22 (63%)	18 (51%)	
Total Positive	28 (80%)			

Activities of subjects to either AMA1 15 mer peptide pools or AMA1 HLA peptide pools are shown; subjects that were negative with both sets of peptide pools were excluded. For each subject, the HLA supertypes that are matched by the tested peptides are italicized in bold and underlined, while predicted promiscuous epitopes are italicized and in bold. Positivity was defined as described in “Methods”

Interpretation (for HLA-typed subjects)

^a^Matched, where activity of an AMA1 HLA pool matched that subject’s HLA

^b^Matched through analysis of promiscuous epitopes in that positive AMA1 HLA pool and that subject’s HLA

^c^HLA-Matched through analysis of epitopes in that positive AMA1 pool that match that subject’s HLA

^d^HLA-Matched through analysis of promiscuous epitopes in that positive AMA1 pool that match that subject’s HLA. For non-HLA-typed subjects

^e^HLA-matched through analysis of epitopes in that positive AMA1 peptide pool that contain matched HLA epitopes

^f^HLA-matched through analysis of promiscuous epitopes in that positive AMA1 peptide pool that contain matched HLA epitopes

^g^In the absence of positive HLA peptide pools, no HLA prediction is possible

### Ex vivo ELISpot IFN-γ responses to predicted HLA-binding AMA1 peptide pools

**Fig. 4:**
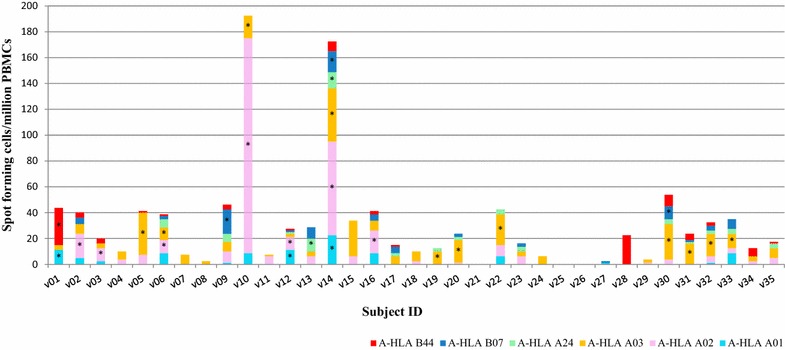
Number of IFN-γ-secreting cells in response to stimulation with the six AMA1 HLA pools. Values are expressed as spot forming cells per million PBMCs. *Stacked bars* represent responses to the different A-HLA pools and *asterisks* indicate responses that met the positivity criteria

Eighteen of the 35 subjects (51 %) responded positively to at least one AMA1 HLA pool (Fig. 4, Table 4). Of these 18 subjects, 13 were positive to one A-HLA pool, four subjects were positive to two A-HLA pools, and one subject (v14) was positive to five of the six A-HLA pools (Fig. 4, Table 4). The highest number of positive responses was to HLA A03 (11 subjects), HLA A02 (seven subjects), followed by HLA B07 and HLA A01 (three subjects each).

Eight of these 18 positive subjects were HLA-typed while the remaining ten subjects were not. Of the eight HLA-typed subjects, six were positive to a single A-HLA pool (v02, v03, v12 and v16 to the A-HLA A02 pool; v05 and v19 to the A-HLA A03 pool), while two subjects were each positive to two different A-HLA pools (v01 to A-HLA A01 and B44 pools; v10 to A-HLA A02 and A03 pools) (Table 4). For the ten subjects with unknown HLA types, seven were positive to a single A-HLA pool (v09 to the A-HLA B07 pool, v13 to the A-HLA A24 pool and v20, v22, v31, v32 and v33 all positive to the A-HLA A03 pool), two were positive to two different A-HLA pools (v06 to the A-HLA A02 and A03 pools; v30 to the A-HLA A03 and B07 pools), and the last subject (v14) was positive to all the six A-HLA pools (restricted by A01, A02, A03, A24, B07, and B44) that were tested (Table 4).

### Interpretation of responses to AMA1 15mer pools and HLA pools

Eleven HLA-typed subjects and 17 non-HLA-typed subjects were positive with either the AMA1 15mer pools or A-HLA pools or both (Table 4). Among the HLA-typed subjects, five were positive with matched A-HLA peptide pools, and three were positive with A-HLA pools that did not match their respective HLA supertypes, though these could be matched through promiscuous (A02) epitopes. This interpretation can be further extended to analysis of activities from three HLA-typed subjects (v04, v08 and v17) who were positive with the AMA1 15mer pools but negative with HLA pools as 15mers within the positive Ap pools contain matching and promiscuous epitopes. Thus both matching and promiscuous epitopes are recognized by these HLA-typed subjects.

For the 17 positive non-HLA-typed subjects, nine subjects were positive with both AMA1 15mer pools and A-HLA pools and eight of these subjects recognized predicted and promiscuous epitopes in the A-HLA pools that match the positive AMA1 15mer pools. This was not the case for the ninth subject (v13) who was positive with the Ap7 and A-HLA A24 peptide pools since there are no predicted HLA A24 epitopes in pool Ap7. The two pools however contain promiscuous epitopes that are restricted by the HLA supertypes A01, A02 and B44. One non-typed subject (v20) was negative with the AMA1 15mer pool but positive with the HLA A03 pool, suggesting that v20 expressed HLA A03. No prediction of the HLA-restriction was possible with the remaining seven subjects that were positive with AMA1 15mer pools but not the A-HLA pools. These outcomes collectively suggest that it is possible to identify the HLA-restriction of responses to AMA1 epitopes in the absence of HLA-typing in 10 of 17 (59 %) non-HLA-typed subjects. Taken together, these results suggest that positive activities of most subjects are detected by both peptide pools spanning regions of CSP or AMA1 or by HLA-specific peptide pools, but epitope promiscuity makes a significant contribution to determining agreement between positive CSP or AMA1 15mer peptide pools and HLA peptide pools.

## Discussion

Development of a malaria vaccine will add to currently available malaria prevention and control tools and greatly enhance ongoing elimination and eradication efforts. Identification of immunodominant T cell epitopes within malaria vaccine target antigens may be important for determining whether malaria vaccines would induce protection in a genetically diverse population. An initial human trial with a DNA-prime adenovirus-boost malaria vaccine using CSP and AMA1 achieved 27 % efficacy [16] and this strategy is being developed further to improve efficacy. Immunity to malaria develops in endemic regions, and is often related to intensity and seasonality of exposure to infected mosquitoes [34]. While naturally acquired immune responses have been better characterized in areas of high transmission [35, 36], only a few studies have shown that natural malaria transmission in areas of lower endemicity also induces significant but low ELISpot activities to antigens such as CSP, CelTOS and AMA1 at these sites [25, 27] and that it is possible to measure these reproducibly using positivity criteria of appropriate stringency that was lower than that used to define high vaccine-induced responses. The aim of this study was to assess 15mer peptides that cover the entire sequences of CSP and AMA1, as well as predicted/known HLA class I-restricted epitopes from the same antigens, for their ability to induce IFN-γ recall responses in PBMCs from naturally exposed individuals in Ghana, and to better determine optimal reagents for further site characterization.

These results show that pools of 15mer peptides spanning the full length of the CSP and AMA1 antigens recall IFN-γ responses in adults, and that these responses are approximately twice as frequent for AMA1 compared to CSP. This confirms and extends data from two previous studies in the same site (Legon) which used ex vivo ELISpot to measure activities to CSP and AMA1 [25, 27]. The first study tested the reproducibility of ex vivo ELISpot in areas of natural malaria transmission, where activities were low, and there were fewer positive responses to CSP than to AMA1 when the same Ap and Cp peptide pools described in the current study were used to stimulate subject PBMCs [25]. Similar observations of relatively lower CSP responses were made in the second study [27] where PBMCs were stimulated with single pools containing all CSP or all AMA1 15mer peptides. It has however been shown in a previous DNA/Ad vaccine trial that single pools containing overlapping peptides that span an entire antigen recall lower ex vivo ELISpot activities than summed activities of multiple peptide pools, each containing fewer peptides [15, 16]. Individual smaller peptide pools are therefore optimal for the characterization of CD8 + T Cell IFN-γ responses in potential vaccine study sites.

The greater activity of AMA1 compared to CSP results from long-term exposure of subjects to AMA1, which is also found in blood stages, during natural malaria transmission [37–40]. In addition, AMA1 (622 amino acids) is larger than CSP (397 amino acids) and is predicted to contain relatively more epitopes and a greater number of immunodominant epitopes compared to CSP (personal observations).

The most frequent responses to CSP were to Cp9 followed by Cp1, and the most frequent responses to AMA1 were to Ap6 followed by Ap1 and Ap2. This differs from earlier observations in Ghana where the most frequent responses were to Cp1, Ap7 and Ap9 [25]. In this earlier study [25], conducted with subjects from Mampong in the Eastern Region of Ghana, smaller CSP HLA peptide pools, or individual HLA peptides, elicited positive responses in 7/19 (36 %) subjects compared to 6/35 (17 %) in this study. It is not known whether this reflects differences in HLA types of the study subjects, or other factors, such as malaria transmission intensity. Mampong, the site of the earlier study, is a semi-urban community about 35 km from Accra [25] and has higher transmission intensity than Legon. A study in a seasonal malaria transmission area in Kenya found no seasonal differences in the frequency of IFN-γ and IL5 ELISpot responses, but higher frequencies of ELISpot responses to IL10 and TNF during the high transmission season compared to the low transmission season [41]. Another study by the same group also found no differences in adult IFN-γ responses between transmission seasons [42]. The role of malaria transmission intensity on the frequency of IFN-γ responses therefore requires further investigation, and future studies may need to factor malaria transmission intensity into site selection for the possibility of identifying additional immunodominant peptides.

Studies using PBMCs from malaria-naïve subjects immunized with adenovirus-vectored AMA1 and CSP candidate vaccines found the highest responses against Cp1, Cp2, Cp6 and Cp9 for CSP [17], and Ap1, Ap3, Ap4, Ap7, Ap8, Ap10 and Ap11 for AMA1 [18]. Thus, positive responses to peptide pools Ap2, Ap6 and Cp4 were observed in the current study but not in studies with malaria-naïve subjects immunized with the gene-based vaccine [16–18]. These differences may be attributable to the genetic (HLA) background of subjects exposed to multiple strains of *P. falciparum* by natural transmission or by vaccine immunization with one strain, 3D7. Subjects in malaria-endemic areas have most likely been repeatedly exposed to a diversity of parasite strains and may thus recognize a broader repertoire of immunodominant peptides. This study only investigated responses to CSP and AMA1 that are undergoing T cell-based vaccine development (16–18). However, T cell epitopes have been identified in other antigens such as LSA1 in Kenya [41, 43] and Madagascar [44], and were associated with a delay in parasitaemia after chemotherapy in Kenya [40]. Similarly, T cell responses to pre-erythrocytic antigens were identified in studies in Gambia [22], and memory responses to TRAP were associated with significantly reduced incidence of malaria [45]. These studies, along with those reported here argue for a more comprehensive investigation of the association of naturally-acquired T cell responses to malaria antigens and resistance to malaria infection and disease. This may be especially true as naturally acquired immunity may result in fewer malaria episodes based on the entomological inoculation rate [6, 46, 47]. However, a previous study in Mali suggested that repeated *P. falciparum* infections do not induce sterile protection [48], suggesting that further studies, especially in sites for likely malaria vaccine trials, are warranted.

It has been suggested that immunodominant T cell epitopes are mostly localized to more conserved regions of AMA1 and that the most polymorphic regions of AMA1 are poorly immunogenic [37]. The most frequent AMA1 pool responses in this study were to Ap6, Ap1 and Ap2; Ap6 contains at least nine polymorphic residues, whereas Ap1 and Ap2 are generally conserved [37]. However, the most polymorphic regions are contained in Ap4 and Ap5 that did not recall responses, in general agreement with the earlier findings [37]. These observations collectively support the need for inclusion of field assessment of antigen peptides in the search for relevant immunodominant peptides for vaccine formulation.

The current study also investigated whether epitope promiscuity was reflected in the epitope-specificity of subjects with natural exposure to *P. falciparum*. Predicted HLA-binding peptide pools, in contrast to the 15mer pools, recalled fewer positive responses than the Cp or Ap pools. This is not a surprise, given that there were much fewer peptides included in the HLA-specific pools (Additional file 1: Table S1 and Additional file 2: Table S2). For subjects that were not HLA-typed, by screening with a series of predicted HLA-binding peptide pools, it was possible to identify putative HLA restrictions for immunodominant epitopes based on the observed and expected ELISpot activities (Tables 3 and 4). A major factor in determining the specificity of these HLA pools was promiscuity of class I-restricted epitopes and this allowed for a more complete interpretation of the activities of the HLA peptide pools, even in the absence of HLA-typing. Similar observations of promiscuity in such epitopes have been reported previously [19, 29, 30]. Promiscuity in class I-restricted epitopes may potentially be important for broad immune coverage as promiscuous epitopes can be recognized by multiple HLA types (Tables 1 and 2). Promiscuity may however be underestimated here since only individual HLA supertypes rather than HLA alleles are included. Studies that aim to identify immunodominant epitopes require HLA-typing of subjects, but these results suggest that it may be possible to use pools of predicted HLA-binding peptides to detect potential immunodominant epitopes without the requirement for HLA-typing and strengthen the argument for inclusion of epitopes from multiple parasite antigens to ensure broad coverage of protective responses in endemic populations [24].

Of the 11 HLA-typed subjects, the HLA supertypes that the predicted epitopes bind to are amongst the most predominant HLA supertypes known globally [32]. However, a better picture of the degree of epitope promiscuity and how this affects class 1-restricted responses to CSP and AMA1 will require HLA-typing of test subjects. HLA typing and testing of a greater number of subjects to assess epitope promiscuity and association of epitope-specific responses with protection from clinical malaria is planned for further studies in Ghana. These results collectively suggest that vaccines containing CSP and AMA1 may induce effective T cell responses in a genetically diverse population.

## Conclusions

In summary, the study has demonstrated the induction of antigen-specific responses in individuals with a history of natural exposure to *P. falciparum*. Peptides in pools Ap1, Ap2, Ap6, Cp1 and Cp9, in addition to other pools that have elicited positive responses from subjects in other studies, therefore require further evaluation. The data also demonstrate the presence of promiscuous HLA class I-restricted epitopes in CSP and AMA1. These epitopes can be recognized by multiple HLA class I types and may thus induce T cell responses in a genetically diverse population, which demonstrates the need to include field assessment of HLA-restricted epitopes in malaria vaccine design strategies. Epitopes identified in these areas will also be useful as standard reagents for the assessment of CD8 + T cell responses in vaccine trials.

## Additional files

10.1186/s12936-016-1098-8 Peptide composition of the CSP 15mer peptide pools.
10.1186/s12936-016-1098-8 Peptide composition of the AMA1 15mer peptide pools.

## Abbreviations

Ad: adenovirus
AMA1: apical membrane antigen 1
Ap: 15mer AMA1 pool
Cp: 15mer CSP pool
C-HLA: predicted CSP HLA-binding 9–10mer peptide pool
A-HLA: predicted AMA1 HLA-binding 9–10mer peptide pool
CSP: circumsporozoite protein
ELISpot: enzyme-linked immunospot assay
HLA: human leukocyte antigen
IFN-γ: interferon-γ
NMIMR: Noguchi Memorial Institute for Medical Research
NMRC: Naval Medical Research Center
PBMCs: peripheral blood mononuclear cells
sfc/m: spot forming cells per million
