# Supplementary material for: Measurement of ex vivo ELISpot interferon-gamma recall responses to Plasmodium falciparum AMA1 and CSP in Ghanaian adults with natural exposure to malaria
Source: Malar J. 2016 Feb 1;15:55. doi: 10.1186/s12936-016-1098-8 (PMC4736649; doi:10.1186/s12936-016-1098-8)
Supplement: Supplementary file 1 — 10.1186/s12936-016-1098-8 Peptide composition of the CSP 15mer peptide pools. [file 12936_2016_1098_MOESM1_ESM.docx]

**Table S1. Peptide composition of the CSP 15mer peptide pools**

| **Pool Number**  **Amino acids** | **Peptide Sequence** | **Predicted HLA epitope** |
| --- | --- | --- |
| **Cp1**  **1-39**  **7 peptides** | **MMRKLAILSVSSFLF** | A02/B27/A24 |
|  | **LAILSVSSFLFV**EAL | B27/A02/A24 |
|  | **SVSSFLFVEALFQEY** | A02/A01/A24 |
|  | F**LFVEALFQEYQCY**G | A01/B27 |
|  | **EALFQEYQCYGSSSN** | A01/B27 |
|  | QE**YQCYGSSSNTR**VL | B27/A03 |
|  | **CYGSSSNTR**VLNELN | A03 |
| **Cp2**  **29-71**  **8 peptides** | SSNTRVLNELNYDNA |  |
|  | RVLNELNYDNAGTNL |  |
|  | ELNYDNAGTNLYNEL |  |
|  | DNAGTNLYNELEMNY |  |
|  | TNLYNELEMNYYGKQ |  |
|  | NELEMNYYGKQENWY |  |
|  | MNYYGKQENWYSLKK |  |
|  | GKQENWYSLKKNSRS |  |
| **Cp3**  **61-107**  **9 peptides** | NWYSLKKNSRSLGEN |  |
|  | LKKNSRSLGENDDGN |  |
|  | SRSLGENDDGNNEDN |  |
|  | GENDDGNNEDNEKLR |  |
|  | DGNNEDNEKLRKPKH |  |
|  | EDNEKLRKPKHKKLK |  |
|  | KLRKPKHKKLKQPAD |  |
|  | PKHKKLKQPADGNPD |  |
|  | KLKQPADGNPDPNAN |  |
| **Cp4**  **97-283**  **12 peptides** | PADGNPDPNANPNVD |  |
|  | NPDPNANPNVDPNAN |  |
|  | NANPNVDPNANPNVD |  |
|  | NVDPNANPNVDPNAN |  |
|  | NANPNVDPNANPNAN |  |
|  | NVDPNANPNANPNAN |  |
|  | NANPNANPNANPNAN |  |
|  | NANPNANPNANPNVD |  |
|  | NANPNANPNVDPNAN |  |
|  | NVDPNANPNANPNKN |  |
|  | NANPNANPNKNNQGN |  |
|  | NANPNKNNQGNGQGH |  |
| **Cp5**  **273-319**  **9 peptides** | NKNNQGNGQGHNMPN |  |
|  | QGNGQGHNMPNDPNR |  |
|  | QGHNMPNDPNRNVDE |  |
|  | MPNDPNRNVDENANA |  |
|  | PNRNVDENANANSAV |  |
|  | VDENANANSAVKNNN |  |
|  | ANANSAVKNNNNEEP |  |
|  | SAVKNNNNEEPSDKH |  |
|  | NNNNEEPSDKHIKEY | A01 |
| **Cp6**  **309-331**  **3 peptides** | E**EPSDKHIKEY**LNKI | A01 |
|  | DKHIK**EYLNKIQNSL** | A02/A24 |
|  | K**EYLNKIQNSLSTEW** | A24/B27 |
| **Cp7**  **321-335**  **6 peptides** | NK**IQNSLSTEW**SPCS | B27 |
|  | NSLSTEWSPCSVTCG |  |
|  | TEWSPCSVTCGNGIQ |  |
|  | PCSVTCGNGIQVRIK |  |
|  | TCGNGIQVRIKPGSA |  |
|  | GIQVRIKPGSANKPK |  |
| **Cp8**  **345-367**  **3 peptides** | RIKPGSANKPKDELD |  |
|  | GSANKPKDELDYAND |  |
|  | KPKDELDYANDIEKK |  |
| **Cp9**  **357-397**  **8 peptides** | ELDYANDIEKKICKM |  |
|  | ANDIEKKICKMEKCS |  |
|  | EKKIC**KMEKCSSVF**N | B27 |
|  | C**KMEKCSSVF**NVVNS | B27 |
|  | KCS**SVFNVVNSSI**GL | A02 |
|  | VF**NVVNSSIGLI**MVL | A02 |
|  | VNSSI**GLIMVLSFL**F | A02 |
|  | SSIG**LIMVLSFLF**LN | B27/B27 |

*P. falciparum* CSP peptide sequence and residue numbers were based on those of the *P. falciparum* clone 3D7 (Gene Bank ID [X15363](http://www.ncbi.nlm.nih.gov/nuccore/X15363)). 15mer peptides were grouped into nine CSP peptide pools. Predicted and known class 1-restricted epitopes within each 15mer peptide are shown in bold and underlined. Since 15mer peptides overlapped by 11 amino acids, class 1 epitopes were often present in more than one 15mer.
