# Supplementary material for: Measurement of ex vivo ELISpot interferon-gamma recall responses to Plasmodium falciparum AMA1 and CSP in Ghanaian adults with natural exposure to malaria
Source: Malar J. 2016 Feb 1;15:55. doi: 10.1186/s12936-016-1098-8 (PMC4736649; doi:10.1186/s12936-016-1098-8)
Supplement: Supplementary file 2 — 10.1186/s12936-016-1098-8 Peptide composition of the AMA1 15mer peptide pools. [file 12936_2016_1098_MOESM2_ESM.docx]

**Table S2. Peptide composition of the AMA1 15mer peptide pools**

| **Pool Number**  **Amino acids** | **Peptide Sequence** | **Predicted HLA epitope** |
| --- | --- | --- |
| **Ap1**  **1-63**  **13 peptides** | MR**KLYCVLLLSA**FEF | A02 |
|  | YCVL**LLSAFEFTY**MI | A01 |
|  | **LLSAFEFTYMI**NFGR | A01/A02/A03/B44 |
|  | F**EFTYMINFGR**GQNY | A03 |
|  | YMINFGRGQNYWEHP |  |
|  | FGR**GQNYWEHPY**QNS | A01 |
|  | Ac-QNYWEHPYQNSDVYR |  |
|  | EHPYQNSDVYRPINE |  |
|  | Ac-QNS**DVYRPINEHR**EH | A03 |
|  | VYRPINEHREHPKEY |  |
|  | INEHREHPKEYEYPL |  |
|  | REHPKE**YEYPLHQEH** | B44 |
|  | KE**YEYPLHQEHTY**QQ | B07/B07 |
| **Ap2**  **53-115**  **13 peptides** | YPLHQEHTYQQEDSG |  |
|  | Ac-QEHTYQQEDSGEDEN |  |
|  | YQQEDSGEDENTLQH |  |
|  | DSGE**DENTLQHAY**PI | B44 |
|  | **DENTLQHAY**PIDHEG | B44 |
|  | LQHAYPIDHEGAEPA |  |
|  | YPIDHEGAEPAPQEQ |  |
|  | HEGAEPAPQEQNLFS |  |
|  | EPAP**QEQNLFSSI**EI | B44 |
|  | Ac-**QEQNLFSSIEIVER**S | B44/A02/A03 |
|  | **LFSSIEIVERSNY**MG | A03/B44 |
|  | **IEIVERSNY**MGNPWT | B44 |
|  | ERS**NYMGNPWTEYM**A | A01/A02 |
| **Ap3**  **105-167**  **13 peptides** | **YMGNPWTEYM**AKYDI | A02 |
|  | PWTE**YMAKYDIEEV**H | A02 |
|  | **YMAKYDIEEV**HGSGI | A02 |
|  | YDIEEVHGSGIRVDL |  |
|  | EVHGSGIRVDLGEDA |  |
|  | SGIRVDLGEDAEVAG |  |
|  | VDLGEDAEVAGTQYR |  |
|  | EDAEVAGTQYRLPSG |  |
|  | VAG**TQYRLPSGK**CPV | A03 |
|  | Ac-QYRLPSGKCPVFGKG |  |
|  | PSGKCPVFGKGIIIE |  |
|  | CPVFGKGIIIENSNT |  |
|  | GKGIIIENSNTTFLT |  |
| **Ap4**  **157-219**  **13 peptides** | IIENSNTTFLTPVAT |  |
|  | SNTTF**LTPVATGNQY** | A01 |
|  | F**LTPVATGNQY**LKDG | A01 |
|  | VATG**NQYLKDGGFAF** | A24 |
|  | N**QYLKDGGFAF**PPTE | A24 |
|  | KDGGFAFPPTEPLMS |  |
|  | FAFPP**TEPLMSPMTL** | B44 |
|  | P**TEPLMSPMTL**DEMR | B44 |
|  | L**MSPMTLDEMRHFY**K | A03/A01 |
|  | M**TLDEMRHFYKDNKY** | A01/A03 |
|  | E**MRHFYKDNKY**VKNL | A01/A03 |
|  | FYKDNKYVKNLDELT |  |
|  | NKYVKNLDELTLCSR |  |
| **Ap5**  **209-271**  **13 peptides** | KNLDELTLCSRHAGN |  |
|  | ELTLCSRHAGNMIPD |  |
|  | CSRHAGNMIPDNDKN |  |
|  | AGNMIPDNDKNSNYK |  |
|  | IPDNDKNSNYKYPAV |  |
|  | DKNSNYKYPAVYDDK |  |
|  | NYKYPAVYDDKDKKC |  |
|  | PAVYDDKDKKCHILY |  |
|  | DDKDKKCHILYIAAQ |  |
|  | KKCHILYIAAQENNG |  |
|  | ILYIAAQENNGPRYC |  |
|  | AAQENNGPRYCNKDE |  |
|  | NNGPRYCNKDESKRN |  |
| **Ap6**  **261-323**  **13 peptides** | RYCNKDESKRNSMFC |  |
|  | KDESKRNSMFCFRPA |  |
|  | KR**NSMFCFRPAK**DIS | A03 |
|  | MFCFRPAKDISFQNY |  |
|  | RPAK**DISFQNYTY**LS | A01 |
|  | **DISFQNYTYLSK**NVV | A01/A03 |
|  | Ac- QNYTYLSKNVVDNWE |  |
|  | YLSKNVVDNWEKVCP |  |
|  | NVVDNWEKVCPRKNL |  |
|  | NWEKV**CPRKNLQNA**K | B07 |
|  | V**CPRKNLQNAK**FGLW | B07 |
|  | KNLQNAKFGLWVDGN |  |
|  | NAKFGLWVDGNCEDI |  |
| **Ap7**  **313-375**  **13 peptides** | GLWVDGNCEDIPHVN |  |
|  | DGNCED**IPHVNEFPA** | B07 |
|  | ED**IPHVNEFPAIDLF** | B07/B44 |
|  | HV**NEFPAIDLF**ECNK | B44 |
|  | FPAIDLFECNKLVFE |  |
|  | DL**FECNKLVFEL**SAS | B44 |
|  | CN**KLVFELSA**SDQPK | A02 |
|  | VFE**LSASDQPKQY**EQ | A01 |
|  | SASDQPKQYEQHLTD |  |
|  | Ac- QPKQYEQHLTDYEKI |  |
|  | YEQHLTDYEKIKEGF |  |
|  | LTDYEKIKEGFKNKN |  |
|  | EKIKEGFKNKNASMI |  |
| **Ap8**  **365-427**  **13 peptides** | EGFKNKNASMIKSAF |  |
|  | NKNASMIKSAFLPTG |  |
|  | SMIK**SAFLPTGAFKA** | A02/A03 |
|  | **SAFLPTGAFKA**DRYK | A03/A03 |
|  | PTGAFKADRYKSHGK |  |
|  | FKAD**RYKSHGKGY**NW | A03 |
|  | **RYKSHGKGYNWGNYN** | A03/A01 |
|  | **HGKGYNWGNY**NTETQ | A01 |
|  | YNWGNYNTETQKCEI |  |
|  | NYNT**ETQKCEIFNV**K | A02/B44 |
|  | E**TQKCEIFNV**KPTCL | A02 |
|  | CEIFNVKPTCLINNS |  |
|  | NVKPT**CLINNSSYI**A | A02 |
| **Ap9**  **417-479**  **13 peptides** | T**CLINNSSYI**ATTAL | A02 |
|  | NNSSYIATTALSHPI |  |
|  | YIATTALSHPIEVEN |  |
|  | TALS**HPIEVENNF**PC | B07 |
|  | **HPIEVENNFPCSLY**K | B07/A01 |
|  | **VENNFPCSLYKDEI**M | A01/B44/B07 |
|  | **FPCSLYKDEIMK**EIE | A03/B07 |
|  | LYKDEIMKEIERESK |  |
|  | EIMKEIERESKRIKL |  |
|  | EIERESKRIKLNDND |  |
|  | ESKRIKLNDNDDEGN |  |
|  | IKLNDNDDEGNKKII |  |
|  | DNDDEGNKKIIAPRI |  |
| **Ap10**  **469-531**  **13 peptides** | EGNKKIIAPRIFISD |  |
|  | KIIAPRIFISDDKDS |  |
|  | PRIFISDDKDSLKCP |  |
|  | ISDDKDSLKCPCDPE |  |
|  | KDSLKCPCDPEMVSN |  |
|  | KCPCDPEMVSNSTCR |  |
|  | DPEMVSNSTCRFFVC |  |
|  | VSNSTCRFFVCKCVE |  |
|  | TC**RFFVCKCVER**RAE | A03 |
|  | FVCKCVERRAEVTSN |  |
|  | CVERRAEVTSNNEVV |  |
|  | RA**EVTSNNEVVV**KEE | A02 |
|  | TSN**NEVVVKEEY**KDE | B44 |
| **Ap11**  **521-583**  **13 peptides** | EVVVKEEYKDEYADI |  |
|  | KEEYKDEYADIPEHK |  |
|  | KDEYADIPEHKPTYD |  |
|  | ADIPEHKPTYDKMKI |  |
|  | EH**KPTYDKMKII**IAS | B07 |
|  | TYDKMKIIIASSAAV |  |
|  | MK**IIIASSAAV**AVLA | A02 |
|  | IASSAAVAVLATILM |  |
|  | AAV**AVLATILMV**YLY | A02 |
|  | **VLATILMVYL**YKRKG | A02/A03 |
|  | **ILMVYLYKRK**GNAEK | A03 |
|  | YLYKRKGNAEKYDKM |  |
|  | RKGNAEKYDKMDEPQ |  |
| **Ap12**  **573-622**  **10 peptides** | AEKYDKMDEPQDYGK |  |
|  | DKMDEPQDYGKSNSR |  |
|  | EPQDYGKSNSRNDEM |  |
|  | YGKSNSRNDEMLDPE |  |
|  | NSRNDEMLDPEASFW |  |
|  | DEMLDPEASFWGEEK |  |
|  | DP**EASFWGEEKR**ASH | A03 |
|  | SFWGEEKRASHTTPV |  |
|  | EEK**RASHTTPVLMEK** | B07/A03 |
|  | **RASHTTPVLMEKPY**Y | A01/B07 |

*P. falciparum* AMA1 peptide sequence and residue numbers were based on those of the *P. falciparum* clone 3D7 (Gene Bank ID 810891). 15mer peptides were grouped into 12 AMA1 peptide pools. Predicted and known class 1-restricted epitopes within each 15mer peptide are shown in bold and underlined. Since 15mer peptides overlapped by 11 amino acids, class 1 epitopes were often present in more than one 15mer.
